# Combination of BCL-2 inhibitors and immunotherapy: a promising therapeutic strategy for hematological malignancies

**DOI:** 10.1007/s12672-024-01161-3

**Published:** 2024-07-26

**Authors:** Xiaohuan Peng, Futian Tang, Yanhong Li, Jun Bai, Lijuan Li, Liansheng Zhang

**Affiliations:** 1 Department of Hematology, The Second Hospital and Clinical Medical School, Lanzhou University, Lanzhou, China; 2 Key Laboratory of the Hematology of Gansu Province, The Second Hospital and Clinical Medical School, Lanzhou University, Lanzhou, China; 3 Key Laboratory of the Digestive Tumor of Gansu Province, The Second Hospital and Clinical Medical School, Lanzhou University, Lanzhou, China

**Keywords:** Hematological malignancies, BCL-2 inhibitor, Immunotherapy, Leukemia

## Abstract

The rapid development of high-throughput sequencing in recent years has facilitated great progress in the molecular-targeted therapy of hematological malignancies, including leukemia, lymphoma, and multiple myeloma. BCL-2 inhibitors are among the most important molecular-targeted agents. Immunotherapy for hematologic malignancy has rapidly increased in popularity in recent years and has been proven to improve the overall survival rate. However, few clinical studies have investigated combination therapy with BCL-2 inhibitors and immunotherapies, such as immune molecule-targeted drugs or immune cell adoptive therapy. In this review, we discuss the drug discovery process, current clinical application status, and resistance and tolerance issues associated with BCL-2 inhibitors. We emphasize their important role in regulating the immune system and propose that the combination of BCL-2 inhibitors with immunotherapy may be one of the most promising treatment methods for hematologic malignancies.

## Introduction

**Fig. 1:**
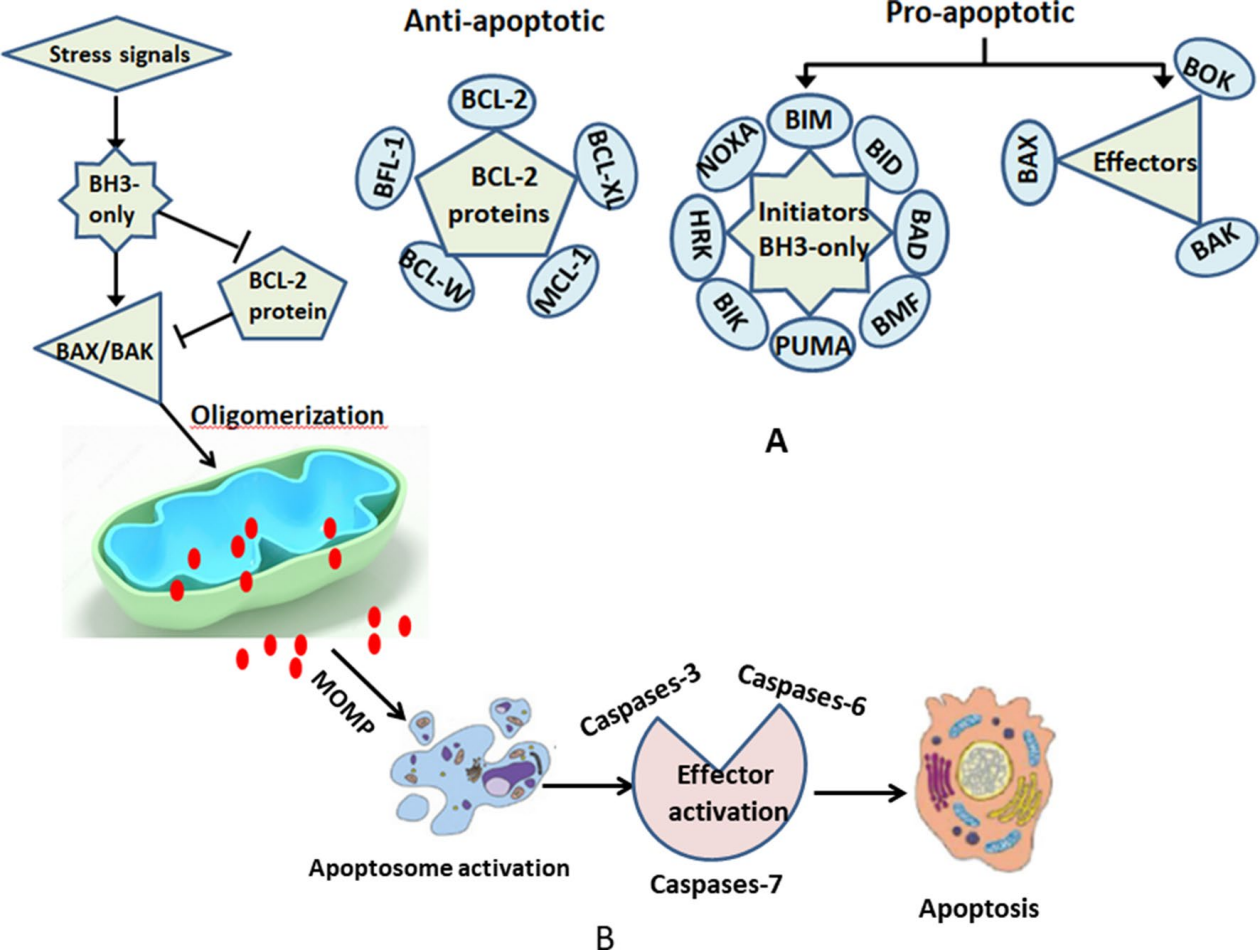
Members of the endogenous apoptosis family (**A**) and the pathways of endogenous apoptosis (**B**)

The rapid development of high-throughput sequencing in recent years has facilitated great progress has in the molecular-targeted therapy of hematological malignancies, including leukemia, lymphoma, and multiple myeloma. BCL-2 inhibitors are among the most important molecular-targeted agents. The BCL-2 antiapoptotic family consists of key molecules that regulate tumor cell apoptosis, including BCL-2, BCL-xL, BCL-w, and MCL-1. They prevent the occurrence of apoptosis by inhibiting the expression of Bak and Bax, thereby inducing tumor cells to evade apoptosis and promoting tumor progression and therapeutic resistance (Fig. 1) [1]. BCL-2 is often overexpressed in hematological malignancies. Therefore, BCL-2 inhibitors are highly promising treatment options and have been proven to be effective and safe [1]. At present, Venetoclax, the first highly selective BCL-2 inhibitor, has been approved by the Food and Drug Administration (FDA) for the treatment of chronic lymphocytic leukemia (CLL) and acute myeloid leukemia (AML) [2].

Immunotherapy, including hemopoietic stem cell transplantation, monoclonal antibodies, immune checkpoint inhibitors and chimeric antigen receptor T-cell (CAR-T cell) immunotherapy, is presently a promising treatment method for eliminating tumor cells. Specifically, immunotherapy plays a powerful antitumor role by enhancing immune function [3]. Some immune agents and adoptive immunocyte therapies have been approved by the FDA for the treatment of hematological malignancies, and emerging antitumor immunotherapies are actively being tested in clinical trials, which have shown enormous therapeutic potential.

Although good treatment results for various hematologic malignancies have been obtained with the use of BCL-2 inhibitors, little is known about how these compounds affect immune cells. Research has shown that Venetoclax can enhance T-cell effector function by increasing reactive oxygen species generation through the inhibition of respiratory chain supercomplex formation, directly increasing their cytotoxicity against AML [4]. F. J. Kohlhapp et al. reported that the combination of Venetoclax and programmed death-1 (PD-1) inhibitors can enhance the killing effect on tumor cells through a dual immune mechanism. Tumor-bearing mice treated with Venetoclax showed an increase in PD-1 + T cells and CD8 + T effector memory cells [5]. A team at the University of Chicago also reported that T cells adapt to BCL-2 inhibitors [6]. Zhigarev et al. reported that venetoclax treatment resulted in a greater fraction of T cells with an effector memory phenotype, inhibited IFN-γ secretion by CD8 + T cells, and downregulated the expression of PD-1 and 2B4 on CD4 + T cells [7]. Researchers have also reported that Venetoclax can enhance the function and antileukemic ability of natural killer (NK) cells and that low-dose Venetoclax has the least impact on NK cells proliferation and viability [8]. Venetoclax can also enhance degranulation and IFN-γ secretion by NK cells, activating NK cells and further enhancing NK cell-mediated antitumor immune effects [8]. These studies indicate that short-term or long-term BCL-2 inhibitor therapy can reshape the body's immune system and enhance the antitumor immune response. Combining immunotherapy with Venetoclax may be a new potential combination for treating hematological malignancies.

Few clinical studies on combination therapy with BCL-2 inhibitors and immunotherapies, such as immune molecule-targeted drugs or immune cell adoptive therapy, are currently available. However, existing studies have confirmed that combination therapy with Venetoclax and previously approved immune molecule-targeted drugs, such as Rituximab, Obinuzumab, and Ibrutinib, was effective and has a long-lasting healing effect [9, 10]. This article reviews the latest clinical research progress on the use of BCL-2 inhibitors in different hematological malignancies and discusses the current status and future prospects of the combination of BCL-2 inhibitors with various new immunotherapies for hematological malignancies.

### BCL-2 family inhibitors

**Fig. 2:**
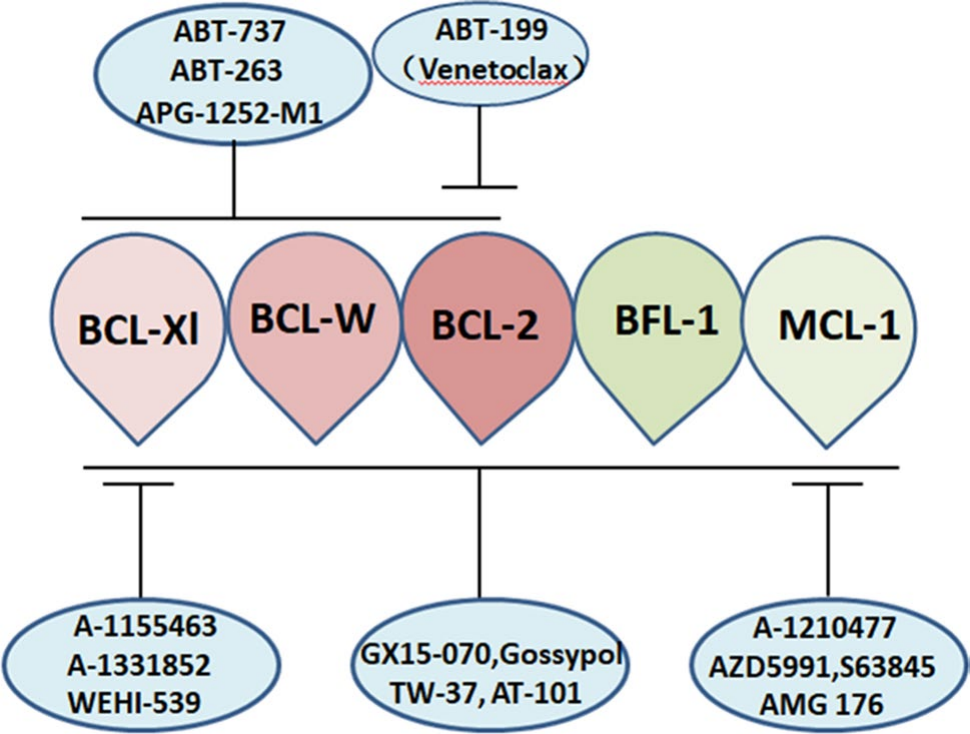
Small-molecule BCL-2 inhibitors for clinical use or evaluation

The BCL-2 gene was first discovered in the chromosomal translocation site (14;18) of follicular lymphoma and was the first mammalian gene associated with cell apoptosis, which subsequently sparked a series of studies on apoptosis and led to the research and development of BCL-2 inhibitors (Fig. 2). Obatoclax is an early pan-BCL-2 inhibitor that can antagonize BCL-2, BCL-xl, BCL-w, and MCL-1 and promote the activation of Bax/Bak and subsequent caspase-3 activation, ultimately leading to the apoptosis of tumor cells. The best response to Obatoclax in a Phase I clinical trial for patients with AML and high-risk myelodysplastic syndrome (HR-MDS) was hematological improvement in 1 AML patient and 3 myelodysplastic syndrome (MDS) patients among 44 hematologic malignancy patients [11]. Obatoclax has poor efficacy but good tolerance, supporting further research on the use of BCL-2 inhibitors in treating hematologic malignancies. The first official BCL-2 inhibitor was ABT-737, which has a high affinity for BCL-2, BCL-xl, and BCL-w and results in significant follicular lymphoma (FL) and CLL cell death in vitro [12]. However, research and development of ABT-737 were ultimately discontinued because of its poor oral bioavailability, which is a serious problem. Navitoclax (ABT-263) is another early BCL-2 inhibitor that overcomes the poor bioavailability of ABT-737 and has shown single-drug antitumor effects on FL and CLL in preclinical and early clinical trials [13]. However, due to the strong inhibitory effect of Navitoclax on BCL-xl, it results in severe dose-dependent thrombocytopenia and ultimately fails to be successfully applied in clinical practice [14]. According to the above studies, researchers have gradually realized that BCL-2-specific inhibitors may become new antitumor drugs for hematological malignancies, and the first highly selective and effective BCL-2 inhibitor, ABT-199 (Venetoclax), was studied in this context. The affinity of Venetoclax for BCL-2 is more than 100 times greater than that for BCL-xl and BCL-w, successfully overcoming the problem of "thrombocytopenia" in clinical applications [15]. Researchers subsequently conducted a series of clinical studies in which Venetoclax was used to treat hematologic malignancies and achieved good results. Thus, Venetoclax was approved for the first-line treatment of elderly AML and CLL patients by the FDA.

The problems of drug tolerance and safety are inevitable in the development and clinical application of all new drugs, and Venetoclax is no exception. Venetoclax has shown good efficacy in patients with AML, relapsed/refractory chronic lymphocytic leukemia (R/R CLL), and relapsed/refractory non-Hodgkin's lymphoma (R/R NHL), but some patients have primary or induced drug resistance problems, leading to ineffective treatment. An in-depth exploration of the drug resistance mechanism of Venetoclax can help solve the problem of resistance and further improve therapeutic efficacy. Venetoclax has high selectivity for BCL-2 but low sensitivity for BCL-xl and MCL-1. Therefore, if tumor cells overexpress BCL-xl and MCL-1, they are resistant to Venetoclax [16]. Studies have shown that resistant AML cell lines have higher transcription levels of MCL-1 and/or BCL-xl and lower BCL-2 than nonresistant AML cell lines [17]. These findings suggest that the antiapoptotic mechanisms of Venetoclax-resistant cells have transitioned from BCL-2-dependent to MCL-1- and/or BCL-xl dependent, especially MCL-1, which prevents BCL-2 inhibitors from inducing tumor cell apoptosis. In addition, previous studies have shown that some gene mutations are involved in Venetoclax resistance. *FLT3-ITD* mutation has been confirmed to indirectly mediate Venetoclax resistance by promoting the expression of MCL-1. The in vitro experimental results also indicate that the *FLT3* inhibitors Midostaurin and Gilteritinib can enhance the antileukemic effect of Venetoclax [18]. *TP53* mutations are also involved in Venetoclax resistance. Nechiporuk et al. reported that *TP53* mutations can help AML cells treated with Venetoclax continue to survive, leading to resistance. [19]. Research has confirmed that the use of murine double minute (MDM) 2 inhibitors to relieve the inhibitory effect of MDM2 on *TP53* can restore the killing effect of Venetoclax on AML cells [20]. In summary, resistance to Venetoclax is one of the most important factors affecting its efficacy and clinical application. If resistance can be detected in a timely manner and overcome by combination therapy, it can benefit patients with hematological malignancies.

Regarding safety, the most common adverse events of Venetoclax are bone marrow suppression, respiratory infections, nausea, vomiting, etc., and severe adverse reactions include tumor lysis syndrome (TLS), agranulocytosis, and invasive fungal infections [21]. TLS is the most serious adverse event associated with Venetoclax, but current research has confirmed that reducing the number of white blood cells and taking allopurinol before using Venetoclax can effectively reduce the incidence of TLS [22]. Agranulocytosis is another serious adverse event of Venetoclax, but the timing of treatment cessation remains debated. Some researchers do not support drug withdrawal and believe that monitoring the complete blood count, the use of antibiotics to prevent serious infection and prompt G-CSF administration will prevent severe agranulocytosis [23]. However, some researchers have also suggested that the dosage should be reduced or that drugs should be stopped when severe granulocyte deficiency occurs [24]. In addition, liver metabolism is easily affected when Venetoclax is combined with other drugs, and the dose should also be appropriately reduced when Venetoclax is combined with P-glycoprotein inhibitors, ciprofloxacin, amiodarone, etc. [21].

After outlining the research and development process, drug resistance, and safety of BCL-2 inhibitors above, we review the current application status of BCL-2 inhibitors in various hematologic malignancies and their combined efficacy with immunotherapy to improve their efficacy.

### Acute myeloid leukemia and high-risk myelodysplastic syndrome

AML and HR-MDS are malignant clonal proliferative disorders whose incidences increase with age. In recent years, the rapid development of molecular-targeted agents and immunotherapy has significantly improved the prognosis of AML and HR-MDS patients. Approximately 70% of AML patients over 65 years of age die within the first year after diagnosis, and the OS of some HR-MDS patients does not exceed one year [25, 26]. Researchers have reported that BCL-2 is overexpressed in some AML and HR-MDS patients and causes treatment resistance, which is associated with poor OS [27, 28]. However, current research has shown that Venetoclax monotherapy has a limited therapeutic effect on AML/HR-MDS, with only a small portion of samples being sensitive in vitro and similar results obtained in clinical trials [29]. In 2016, a Phase II single-arm clinical trial showed that the objective response rate (ORR) of Venetoclax monotherapy for AML was only 19% [30]. Another Phase II clinical trial revealed that the median OS of patients receiving Venetoclax monotherapy for relapsed and refractory HR-MDS was only 5 months [31]. However, Venetoclax combined with hypomethylating agents (HMAs) has shown significant antitumor activity in AML/HR-MDS. A nonrandomized, open-label, Phase Ib study reported a median OS of 16.9 months in elderly patients with previously untreated AML treated with Venetoclax combined with HMAs [32]. The results in HR-MDS patients are similar to those in AML patients. A meta-analysis revealed that the overall response rate of Venetoclax combined with azacitidine in the treatment of newly diagnosed HR-MDS was 61%, and the 18-month OS rate was 74% [33]. The FDA has approved Venetoclax in combination with HMAs or low-dose cytarabine for older adults with newly diagnosed AML [34]**,** and this regimen has been proven to be effective in Phase III, randomized, placebo-controlled clinical trials [34].

However, the efficacy of Venetoclax combined with HMAs in the treatment of relapsed/refractory AML/HR-MDS is limited. Aldoss et al. retrospectively analyzed the outcomes of 90 adults with relapsed/refractory acute myeloid leukemia (R/R AML) who were treated with Venetoclax combined with HMAs and reported that the rate of complete remission (CR)/CR with incomplete hematologic recovery (CRi) was only 46% [35]. The total ORR of 18 HR-MDS patients from the United States receiving Venetoclax combined with HMA treatment was 44%, and the median OS was 11.4 months [36]. Therefore, new combined treatment regimens need to be studied to improve the curative effect of R/R AML/HR-MDS. Research has shown that combining Venetoclax with monoclonal antibodies and T-cell-based immunotherapy elicits synergistic cytotoxicity in vitro, indicating that the combination of Venetoclax and immunotherapy may have synergistic antitumor effects on the induction of cell lysis [37].

NK cells, which are at the forefront of tumor monitoring, play an important role in the immunotherapy of AML/HR-MDS. NK cell-based immunotherapies with targeted agents or bi- and trispecific killer engagers (BiKEs and TriKEs, respectively) have been confirmed to produce combined effects [38]. Therefore, the synergism between NK cell-based immunotherapy and the BCL2 inhibitor Venetoclax has attracted the attention of scholars. Researchers have shown that Venetoclax can enhance the function and antileukemic ability of NK cells and that low-dose Venetoclax has the least impact on NK cell proliferation and viability [8]. This study revealed that Venetoclax can upregulate the expression of the NK cell receptor (NKG2D) and induce the expression of ligands to the corresponding protein (NKG2DL), playing a bidirectional immune regulatory role between NK and AML cells, activating the NKG2D/NKG2DL immune signaling pathway, and ultimately enhancing its antileukemic effect. Venetoclax can also increase degranulation and interferon-γ (IFN-γ) secretion by NK cells, activating NK cells and further enhancing NK cell-mediated antitumor immune effects [8]. Overall, combination therapy with Venetoclax and NK cell-based immunotherapy has shown stronger antitumor effects in vitro.

CD3 + CD4-CD8 double-negative T cells (DNTs) are a rare and unique subset of T cells with strong antileukemia capacity that selectively target AML cells without causing toxicity to normal cells or tissues [39]. Lee JB et al. reported that Venetoclax can effectively enhance the antitumor function of DNT cells and CD8 + T cells by increasing the generation of reactive oxygen species (ROS) but does not reduce the number of T cells [4, 40]. In addition, combination with HMAs improves efficacy. Based on the above research, we believe that Venetoclax can be used as an immunomodulatory drug and that combination therapy with adoptive T-cell therapy and Venetoclax may be a promising combination therapy for AML patients, which may provide new hope for R/R AML patients [7]. However, further research is needed.

Leukemic stem cells (LSCs) are considered responsible for the development, progression, drug resistance and recurrence of leukemia. CD123 is a specific antigen on LSCs, and antibody drugs targeting CD123 can effectively kill LSCs in AML patients, significantly improving their prognosis. IMGN632 is a CD123-targeting antibody‒drug conjugate and is currently being studied in AML. Preliminary studies have suggested that the combination of azacitidine, Venetoclax, and IMGN632 can synergistically induce AML cell death. A Phase Ib/II clinical study of IMGN632 combined with Venetoclax is currently underway (NCT0486264) [41]. CD33 is another suitable target for the targeted treatment of AML. CD33-targeting antibodies, such as Gemtuzumab ozogamycin, are promising new drugs for the treatment of AML and can significantly improve the 2-year OS rate. The Phase Ib/II clinical trial (NCT03390296) of Gemtuzumab ozogamicin combined with Venetoclax for newly diagnosed AML achieved good efficacy; the CR/CRi rates were 50%, and the 1-year OS rate was 31% [42, 43]. 225 Actinium lintuzumab (225Ac lintuzumab) is a radioimmunotherapy in the clinical stage of development that targets CD33 and has been shown to have monotherapy activity in R/R AML. Research has shown that, compared with the use of 225Ac lintuzumab and Venetoclax alone, combined therapy synergistically induces tumor cell killing effects in Venetoclax-resistant AML cell lines, significantly inhibiting tumor growth and prolonging survival [44]. This treatment strategy may be promising for R/R AML patients, and clinical trials are currently underway (NCT03867682). Research has confirmed that AML/HR-MDS cells overexpress CD47; therefore, blocking the CD47/SIRPα signaling pathway has a strong antileukemic effect. Consequently, anti-human CD47 monoclonal antibodies are promising immunotherapy drugs for AML/HR-MDS [45]. A Phase Ib/II clinical trial of Venetoclax combined with HMAs and Magrolimab, which is a new anti-human CD47 monoclonal antibody, showed promising results in the treatment of R/R AML and de novo AML, but the trial investigating this combination was limited by futility [42, 46]. Preliminary results revealed that the CR + CRi rates for *TP53*-mutated and *TP53* wild-type AML patients were 63% and 86%, respectively [46]. Evorpacept (ALX148), a CD47-blocking myeloid checkpoint inhibitor, was tested in combination with Venetoclax and azacitidine in R/R AML or de novo AML patients. An objective response was observed in all de novo AML patients and 44.4% of R/R AML patients [47]. Cusatuzumab is a high-affinity, anti-CD70 monoclonal antibody that has shown preliminary activity in AML, and a Phase Ib study investigating the triple combination of Cusatuzumab, Venetoclax and azacitidine is underway (NCT04150887) [48].

As an emerging treatment method, the combination of a BCL-2 inhibitor and an immune checkpoint inhibitor may be a new potential therapy for HR-MDS/AML patients, and corresponding clinical research is currently underway (NCT03390296) [42, 43]. At present, clinical trials of BCL-2 inhibitors combined with immunotherapy in HR-MDS/AML patients are rare. However, current basic medical research has confirmed that BCL-2 inhibitors combined with immunotherapy may be promising therapies for HR-MDS/AML patients (Table 1), but further research is needed.

**Table 1:** Clinical trials of the combined application of BCL-2 inhibitors and immunotherapy in AML patients and HR-MDS patients

Therapeutic regimen	Immune targets	Population	NCT	Phase	Response	Main adverse events	References
VEN + IMGN632 + Aza	CD123	R/R AML	NCT0486264	Ib/II	CR + CRi rates was 31%	CRS	[41]
VEN + Aza + GO	CD33	De novo AML	NCT03390296	I/II	median OS was 12.8 months	Febrile neutropenia	[42, 43]
VEN + 225 Ac lintuzomab	CD33	R/R AML	NCT03867682	I/II	Recruiting	Recruiting	[44]
VEN + Magrolimab	CD47	R/R AML and de novo AML	NCT04435691	Ib/II	Unpublished	Unpublished	[42]
VEN + Aza + Magrolimab	CD47	R/R AML and de novo AML	NCT05079230	Ib/II	CR + CRi rates for *TP53* wild-type patients were 86%	Febrile neutropenia, pneumonia	[46]
VEN + Aza + Evorpacept	CD47	R/R AML and de novo AML	NCT03013218	I	ORR in de novo AML was 100% and R/R AML was 44.4%	One patient experienced CRS	[47]
VEN + Aza + Cusatuzumab	CD70	De novo AML	NCT03030612	I	Unpublished	Unpublished	[48]
VEN + Aza + Avelumab	PD-L1	De novo AML	NCT03390296	I/II	The median OS was 4.8 months	Infection, Febrile neutropenia	[42, 43]

*R/R AML* relapsed/refractory acute myeloid leukemia, *VEN* Venetoclax, *Aza* Azacytidine, *GO* Gemtuzumab ozogamicin, *CR + CRi* complete remission (CR)/CR with incomplete hematologic recovery (CRi), *OS* overall survival, *ORR* overall response rate, *CRS* cytokine release syndrome

### Chronic lymphocytic leukemia

CLL is a clonal malignant disease of lymphocytes. The treatment of CLL has undergone a transition from chemotherapy to small-molecule targeted therapy and cellular immunotherapy, including novel CD20 monoclonal antibodies, Bruton’s tyrosine kinase (BTK) inhibitors, phosphatidylinositol 3-kinase inhibitors and CAR-T-cell treatment [49]. At present, single-drug treatment for CLL has excellent efficacy, but some patients still need to explore new drugs or combination therapy options due to drug resistance and recurrence. Research has confirmed that BCL-2 is highly expressed in CLL cells and that most CLL cells rely on BCL-2 for survival. Therefore, BCL-2 inhibitors can effectively promote CLL cell apoptosis, and the FDA has approved Venetoclax for the treatment of adult CLL. In a Phase II, single-arm, multicenter study, the ORR of Venetoclax monotherapy for del(17p) CLL patients was 80% (NCT01889186) [50]. Another clinical trial also revealed that Venetoclax monotherapy had an ORR of 71% and a PR rate of 64% in R/R CLL patients (NCT01328626) [51]. However, the efficacy of Venetoclax monotherapy remains limited for some R/R CLL patients, and CLL cells accumulate unfavorable biological characteristics during treatment, gradually developing drug resistance.

In the last twenty years, immunotherapy involving anti-CD20 monoclonal antibodies has significantly improved the prognosis of CLL patients, and new immunotherapies, such as immune checkpoint inhibitors, bispecific and trispecific antibodies, CAR-T-cell therapy, and NK-cell adoptive therapy, have also shown impressive results in some relapsed and refractory CLL (R/R CLL) patients.

Chimeric anti-CD20 monoclonal antibodies (mAbs) are the most widely used immunotherapies in clinical practice for CLL patients, completely changing the treatment strategy for CLL. Rituximab is the earliest anti-CD20 mAb that can significantly increase the CR and ORR rates in CLL patients and improve their progression-free survival (PFS) and OS [52]. The therapeutic effect of Rituximab as a single drug in CLL is limited, and some patients remain unsuccessful in achieving remission [53]. Therefore, combined treatment seems to improve the efficacy of Rituximab. BCL-2 inhibitors are currently among the most popular molecular-targeted and combination drugs for CLL patients (Table 2). An early Phase II clinical trial revealed that in newly diagnosed CLL patients, the ORR of Rituximab combined with the BCL-2 inhibitor Navitoclax was 70%, whereas the ORR of Rituximab alone was 35% (NCT01087151) [54]. The Phase Ib M13-365 trial released in early 2017 was the first study to officially validate the efficacy of Venetoclax combined with Rituximab (VR) in R/R CLL. This study revealed that the VR regimen achieved 51% CR, 82% PFS, and 89% sustained response in R/R CLL patients, which is much greater than the rates associated with the individual uses of the two drugs (NCT01682616) [55]. The international randomized MURANO clinical trial comparing the VR regimen with the standard immunochemotherapy regimen further confirmed the efficacy of VR combination therapy. This clinical trial revealed that, compared with that of patients receiving 6 cycles of immunochemotherapy with Bendamustine plus Rituximab (BR), the efficacy of VR was more satisfactory, with a 24-month PFS of 84.9% and an OS of 91.9%, whereas BR had a PFS of 36.3% and an OS of 86.6% (NCT02005471) [56]. In the latest report of this clinical trial, the median PFS of VR was 53.6 months, that of BR was 17 months, and the 5-year OS rates for VR and BR were 82.1% and 62.2%, respectively [57]. Moreover, adverse reactions did not significantly differ between the two groups.

**Table 2:** Clinical trials of the combined application of BCL-2 inhibitors and immunotherapy in CLL patients

Therapeutic regimen	Immune targets	Population	NCT	Phase	Response	Main adverse events	Reference
ABT263 + Rituximab	CD20	De novo CLL	NCT01087151	II	ORR rate 70%	neutropenia, gastrointestinal symptoms	[54]
Ven + Rituximab	CD20	R/R CLL	NCT01682616	Ib	CR rate 51%, 24-month PFS rate 82%	Neutropenia and infections	[55]
Ven + Rituximab	CD20	R/R CLL	NCT02005471	III	24-month PFS rate 84.9%, OS rate 91.9%	Neutropenia and infections	[56]
VEN + Ofatumumab	CD20	De novo CLL	NCT02242942	Ib	CR rate 58%, 15-month PFS rate 100%	Neutropenia and infections	[60]
VEN + Obinutuzumab	CD20	De novo CLL and R/R CLL	NCT01685892	1b	ORR rate 95% (R/R) and 100% (de novo)	Neutropenia and infections	[61]
VEN + Obinutuzumab	CD20	De novo CLL	NCT02242942	III	24-month PFS rate was 88.2%	Neutropenia and infections	[63]
VEN + Ibrutinib	BTK	R/R CLL	ISCRTN13751862	II	CR rate 51%	neutropenia or gastrointestinal adverse events	[67]
VEN + Ibrutinib	BTK	R/R CLL	NCT02756897	II	12-month PFS after suspension of the drug rate was 98%	Infections, neutropenia and gastrointestinal adverse events	[68]
VEN + Ibrutinib	BTK	De novo CLL	NCT02910583	II	24-month PFS and OS rates were 95% and 98%	Neutropenia and hypertension	[69]

*R/R CLL* relapsed/refractory chronic lymphocytic leukemia, *VEN* Venetoclax, *BTK* Bruton’s tyrosine kinase, *CR* complete remission, *OS* overall survival, *ORR* overall response rate, *PFS* progression-free survival

The combination of Venetoclax and Rituximab has indeed changed the treatment mode of CLL and has achieved success, but some patients do not respond to Rituximab. To further prolong survival, a new generation of anti-CD20 mAbs, such as Ofatumumab, has entered the stage of CLL treatment. Ofatumumab is the first Type I anti-CD20 mAb. Compared with Rituximab, it has stronger complement-dependent cytotoxicity and more stable binding with CD20, showing good efficacy and tolerance in R/R CLL patients [58]. Obinutuzumab (GA101) is a humanized, monoclonal Type II anti-CD20 mAb, and clinical trials have confirmed that obinautuzumab monotherapy has good safety and efficacy in treating R/R CLL patients [59]. In summary, the above studies indicate that Ofatumumab/Obinutuzumab is an effective drug for CLL, and the development of combination therapy consisting of Ofatumumab/obinutuzumab and Venetoclax is warranted. In 2017, Fischer K et al. first evaluated the combined effect of a combined Venetoclax and obinutuzumab regimen in R/R CLL and reported that the three-month ORR was 100%, with 7 out of 12 patients experiencing CR [60]. A series of Phase Ib and II clinical trials subsequently confirmed the excellent efficacy and tolerability of the combined Venetoclax and Obinutuzumab regimen in the early stage. The overall response rate was 95% in R/R CLL patients and 100% in newly diagnosed patients. The most common Grade 3–4 adverse events were neutropenia, and TLS did not occur [10, 61, 62]. CLL14 is an open, randomized, Phase III trial targeting previously untreated CLL patients. The ORR and CR rates of the Venetoclax plus Obinutuzumab group were significantly greater than those of the Chlorambucil plus Obinutuzumab group (Table 3) [63, 64]. The above studies further confirmed that the combination of Venetoclax and a CD-20 mAb is a more effective therapeutic and safe for both treatment-naive CLL and R/R CLL patients and may become a new ideal treatment option for CLL patients in the future.

**Table 3:** Clinical trials of the combined application of BCL-2 inhibitors and immunotherapy in MM patients

Therapeutic regimen	Immune targets	Population	NCT	Phase	Response	Main adverse events	Reference
VEN + Pomalidomide	Immunomodulators	R/R MM	NCT03567616	II	median PFS was 10.5 months	Neutropenia	[86]
VEN + Daratumumab	CD38	R/R MM	NCT03314181	I	ORR rate was 96%, 18-month and PFS rate was 90.5%	Diarrhea and nausea	[91]
VEN + Daratumumab	CD38	R/R MM	retrospective study	-	ORR rate of t(11;14) R/R MM was 80%	Gastrointestinal adverse events	[92]

*R/R MM* relapsed/refractory multiple myeloma, *VEN* Venetoclax, *OS* overall survival, *ORR* overall response rate, *PFS* progression-free survival

Bruton's tyrosine kinase (BTK) is a critical kinase in the B-cell receptor (BCR) signaling pathway and is involved in the proliferation, differentiation, and apoptosis processes of B cells. BTK inhibitors such as Ibrutinib can inhibit the malignant proliferation of B-cell tumors, induce cell apoptosis, and reduce the secretion of chemokines and inflammatory factors, ultimately producing immunotherapeutic effects [65]. Previous preclinical studies have shown that BTK inhibition enhances mitochondrial BCL-2 dependence, thereby enhancing the killing effect of BCL-2 inhibitors [66]. Therefore, combination therapy with Ibrutinib and Venetoclax may have potential synergistic effects on CLL patients. The CLARITY study (ISCRTN13751862) revealed that 51% of R/R CLL patients achieved a CR after 12 months of treatment with Ibrutinib plus Venetoclax. Moreover, the peripheral blood of 53% of patients and the BM of 36% of patients was free of residual disease, and treatment only resulted in mild and controllable adverse reactions [67]. HOVON141/VISION (NCT02756897) was an open-label, randomized, Phase II trial released in 2022 that evaluated the efficacy of Ibrutinib combined with Venetoclax in R/R CLL. Twelve months after discontinuation, the PFS was 98%, and the common adverse events were infection (58%), neutropenia (40%), and gastrointestinal adverse events (24%) [68]. CAPTIVATE (NCT02910583) is an international Phase II study that revealed that the combination of Ibrutinib and Venetoclax treatment had a 55% CR rate, 95% 24-month PFS rate and 98% 24-month OS rate in newly diagnosed elderly CLL patients. The most common adverse events were neutropenia (33%) and hypertension (6%) [69]. In addition, that study revealed that combination therapy with these two drugs can significantly reduce the risk of Venetoclax related TLS- and Ibrutinib-related hemorrhages [69, 70]. In addition, Skånland SS et al. confirmed that reducing the standard doses of Ibrutinib plus Venetoclax treatment can reduce drug costs and toxicity but does not affect efficacy [71]. In summary, combination therapy with Ibrutinib plus Venetoclax has achieved promising results in clinical studies and can reduce the toxicity of these drugs, with good tolerability.

T-cell-based immunotherapy, bispecific antibodies, immune checkpoint inhibitors, and tumor vaccines are important components of tumor immunotherapy, but reports on the combination of Venetoclax and these immunotherapies for CLL patients are scarce. Murakami S et al. first discovered that combining Venetoclax with cytomegalovirus (CMV) pp65 antigen-specific cytotoxic T cells (CMV-CTLs) improved the therapeutic effect on CMVpp65-transfected B-cell tumor compared to monotherapy, indicating that Venetoclax and CMV-CTLs have synergistic cytotoxic effects. This study suggested that T-cell-based immunotherapy combined with Venetoclax is effective against B-cell malignancies [37]. CAR-T cells are new therapeutic T cells that can specifically kill tumor cells. Studies have shown that taking Venetoclax before CAR-T-cell therapy can upregulate the expression of CD19 and proapoptotic proteins in B-CLL cells, resulting in increased cytotoxicity and the persistence of CD19 CAR-T cells [72]. This regimen is an exciting new strategy but needs to be validated in future in vivo research and clinical trials. In addition, current research suggests that the poor efficacy of immune cell-based immunotherapy in CLL may be related to T-cell dysfunction [73]. Venetoclax has been proven to significantly improve the immune function of T lymphocytes, thereby enhancing the efficacy of T-cell-based immunotherapy [74]. Future research should focus on the efficacy of Venetoclax combined with T/NK cell-based immunotherapy in the treatment of R/R CLL.

### Multiple myeloma

Multiple myeloma (MM) is an abnormal clonal proliferative disease of plasma cells and is the second most common hematological malignancy. In the past 20 years, the treatment of MM has made continuous progress, but MM remains incurable, and most patients still experience problems with recurrence. Research has shown that the bone marrow microenvironment of MM can induce high expression of the antiapoptotic BCL-2 family, thereby promoting the survival of MM cells [75]. In addition, approximately 20% of MM patients carry chromosome t(11;14)-positive translocations associated with high BCL-2 and low MCL-1/BCL-xl expression. Furthermore, preclinical data indicate that myeloma cell lines and primary myeloma samples with t(11;14)-positive translocations are highly sensitive to Venetoclax [76]. Therefore, Venetoclax may be an attractive therapeutic agent for MM patients, especially those with t(11; 14) translocations.

In recent years, Venetoclax-based clinical trials have achieved remarkable results in R/R MM. An early Phase Ia clinical trial revealed that the ORR of Venetoclax monotherapy for R/R MM was 21%, with an ORR of up to 40% in MM patients with the t(11; 14) translocation (NCT01794520). The most common adverse reactions were gastrointestinal toxicity and thrombocytopenia [77]. A Phase I clinical trial reported by Touzeau C et al. further confirmed that Venetoclax monotherapy has good efficacy in treating R/R MM patients [78]. In addition, other antimyeloma drugs have been shown to regulate BCL-2 expression. For example, Dexamethasone treatment can promote Bcl-2 dependence in MM, resulting in increased sensitivity to Venetoclax [79]. Furthermore, Bortezomib and Carfilzomib can reduce Venetoclax resistance, which is mediated by MCL-1 [80]. Therefore, we believe that combination therapy based on Venetoclax may be more effective than monotherapy in MM patients. In addition to MM patients, Venetoclax alone (400 mg) has been used to treat R/R light chain amyloidosis patients, which resulted in an ORR of up to 66.6% [81]. Moreover, reports on Venetoclax therapy in plasma cell leukemia patients are mostly case reports, but they also show good results. In one case, a combination regimen consisting of daratumumab, bortezomib, Venetoclax, and dexamethasone induced a rapid and strong hematologic response [82].

Immune dysfunction is an important factor that promotes the occurrence and development of MM. Therefore, immunotherapy has great potential for the treatment of MM. BCL-2 inhibitors, such as Venetoclax and immunotherapy, are efficacious in the treatment of R/R MM (Table 3). Therefore, the combination of these two methods to further improve their therapeutic effect in R/RMM patients has attracted the attention of many scholars, and several clinical trials have been conducted. Immunomodulators such as Thalidomide, Lenalidomide, and Pomalidomide have been approved by the FDA for the treatment of MM and have also been approved by the National Comprehensive Cancer Network guidelines as first-line treatments for MM [83, 84]. The combination immunomodulators and the second-generation proteasome inhibitor Carfilzomib is increasingly being used as induction therapy in high-risk MM patients and R/R MM patients [85]. A Phase II clinical trial (NCT03567616) evaluated the efficacy of Venetoclax combined with Pomalidomide and Dexamethasone in R/R MM. Sixty-three percent of R/R MM patients achieved remission, with a median remission time of 12.9 months and a median progression-free survival time of 10.5 months [86]. Sidiqi MH et al. subsequently reported that, compared to the Venetoclax monotherapy, combination therapy with Lenalidomide/ Pomalidomide had a median PFS of 5.8 months and a median OS of 28.4 months. in R/R MM patients [87]. These findings confirm that the combination of Venetoclax and immunomodulators may improve the prognosis of R/R MM patients, but further research is needed.

CD38 is a Type II transmembrane glycoprotein highly expressed on the surface of MM cells and is another ideal therapeutic target for MM. Anti-CD38 mAbs can promote MM cell death through immune mechanisms, such as complement-dependent cytotoxicity, antibody-dependent cellular cytotoxicity, antibody-dependent cellular phagocytosis, amplification of effector T cells, and a reduction in regulatory T cells [88]. Currently, three anti-CD38 mAbs have been developed in clinical practice: daratumumab, Isatuximab and MOR202. Among them, Daratumumab is the first fully human mAb to target CD38 and has become the most important medication for R/R MM. Clinical trials of Daratumumab monotherapy have preliminarily verified its safety and effectiveness, and combining Daratumumab with other drugs accelerates and strengthens remission (NCT00574288 and NCT01985126) [89]. In as early as 2018, Rahbari KJ et al. used a combination treatments regimen consisting of Daratumumab, Dexamethasone, Venetoclax, and Bortezomib to treat 2 patients with R/R MM. Both patients achieved rapid and sustained CR without significant adverse reactions [90]. This study is the first report of the use of Daratumumab combined with Venetoclax for the treatment of R/R MM. Subsequently, Bahlis NJ et al. conducted a Phase I clinical study (NCT03314181) to evaluate the efficacy of Venetoclax combined with Daratumumab and Dexamethasone in R/R MM patients with t(11; 14) translocations and reported an ORR of 96% and an 18-month PFS of 90.5% [91]. These results support the continued evaluation of the efficacy and safety of the Venetoclax combined with Daratumumab in the treatment of R/R MM patients, especially patients with t(11; 14) translocations. When Venetoclax combined with daratumumab was used to treat R/R MM, > 400 mg/day Venetoclax lead to serious adverse events, such as infections. Therefore, the efficacy of low-dose Venetoclax combined with Daratumumab in R/R MM has become another research hotspot [92]. In 2021, Regidor B et al. conducted a retrospective study on the efficacy and safety of low-dose Venetoclax (≤ 250 mg/day) combined with Daratumumab and Dexamethasone in the treatment of R/R MM. This study revealed that low-dose Venetoclax treatment resulted in reduced efficacy among those lacking t(11;14) translocations (ORR = 31%), but patients harboring the t(11;14) marker exhibited an ORR of 80% and did not experience serious adverse events, such as frequent infection [93]. This study demonstrated that low-dose Venetoclax also has good therapeutic effects on R/R MM patients with the t(11;14) marker. Therefore, the dose can be appropriately reduced for some patients who do not tolerate the 400 mg dose. In summary, the results of these studies indicate that adding Daratumumab to the Venetoclax-based regimen is safe and can generate strong and long-lasting treatment responses; this treatment strategy may have synergistic effects, supporting further exploration of Venetoclax combined with anti-CD38 immunotherapy to treat R/R MM. In addition, studies have shown that Venetoclax plus Daratumumab can enhance antibody-dependent cell-mediated cytotoxicity (ADCC)-induced NK cell cytotoxicity in MM cell lines, possibly through the activation of the mitochondrial apoptosis pathway [93]. These findings suggest that Venetoclax combined with Daratumumab and NK cell adoptive therapy may be promising treatment options for R/R MM patients.

### Non-Hodgkin's lymphoma

Non-Hodgkin's lymphoma (NHL) is one of the most common hematological malignancies, accounting for nearly 3% of cancer diagnoses and deaths [94]. In recent years, the emergence of new targeted drugs and immunotherapies has significantly improved the efficacy of NHL treatment. Research has revealed that some NHL patients, especially B-cell lymphoma patients, overexpress BCL-2, which promotes tumor cell resistance to chemotherapy drugs and is associated with poor prognosis [95]. Therefore, BCL-2 inhibitors may be good treatment options for these patients and may improve patient prognosis.

Venetoclax has shown great potential in the treatment of NHL. The expression of BCL-2 varies greatly among different types of NHL, and the therapeutic effects consequently also differ greatly. Phase I clinical trials have confirmed that mantle cell lymphoma (MCL) patients are sensitive to Venetoclax monotherapy, with an ORR of 75% in R/R MCL patients and a median PFS of 14 months [96]. Studies have shown that R/R FL patients have high levels of BCL-2, but the response rate of these patients to Venetoclax monotherapy is not ideal, with an ORR of only 38%, a CR of 21%, and a median PFS of only 11 months [96]. Diffuse large B-cell lymphoma (DLBCL) is the most common type of malignant lymphoma, accounting for 30%-40% of NHLs. Research has shown that BCL-2 expression is highly heterogeneous in DLBCL patients, and Venetoclax monotherapy consequently has poor efficacy in some RR-DLBCL patients, with an ORR of only 38% and a CR of 12% [96]. Therefore, the activity of and tolerance to the Venetoclax monotherapy vary by NHL subtype, and combination therapy may improve the response rate and tolerance.

Immunotherapy has been increasingly applied to treat lymphoma, especially NHL. Immunotherapy, including anti-CD20 mAbs, immunomodulators, immune checkpoint inhibitors, BTK inhibitors, and CAR-T-cell immunotherapy, has been effective. Because Venetoclax and immunotherapy are efficacious in NHL patients, the combined effect of these two agents has attracted the attention of scholars, leading to several studies (Table 4). Specifically, studies have examined effectiveness and safety of anti-CD20 mAbs plus Venetoclax. The CONTALTO clinical trial conducted by Zinzani PL et al. evaluated the safety and efficacy of Venetoclax combined with Rituximab in R/R FL patients. However, the results showed poor efficacy, with a CR of 17% (NCT02187861) [97]. Obinutuzumab is a second-generation anti-CD20 mAb. Stathis A et al. conducted a Phase I study to evaluate the safety, tolerability, and initial efficacy of Obinuzumab combined with Venetoclax in treatment-naive FL patients. They reported that the ORR was 87.5%, the one-year PFS rate was 77.8%, and neutropenia was the most common adverse event (NCT02877550) [98].

**Table 4:** Clinical trials of the combined application of BCL-2 inhibitors and immunotherapy in NHL patients

Therapeutic regimen	Immune targets	Population	NCT	Phase	Response	Main adverse events	Reference
Ven + Rituximab	CD-20	R/R FL	NCT02187861	II	CR rate was 17%	Neutropenia and thrombocytopenia	[97]
VEN + Obinutuzumab	CD-20	De novo FL	NCT02877550	I	ORR was 87.5%, one-year PFS rate was 77.8%	Neutropenia	[98]
VEN + Ibrutinib	BTK	MCL	NCT02471391	II	CR rate was 71%	Diarrhea, fatigue and nausea	[100]
VEN + Ibrutinib	BTK	R/R MCL	NCT03112174	III	median ORR was 81% and CR rate was 62%	Infections, diarrhea, neutropenia	[101]
VEN + Ibrutinib	BTK	R/R MCL	NCT02419560	I/Ib	ORR was 82.3%, CR rate was 42.4%	Neutropenia, diarrhea	[103]
VEN + RO6870810 + Rituximab	BTK CD20	DLBCL	NCT03255096	Ib	Total effective rate was 38.5%, and the CR rate was 20.5%	Neutropenia, anemia and thrombocytopenia	[106]
VEN + Ibrutinib + Rituximab	BTK CD20	R/R DLBCL	NCT03136497	Ib	median PFS of 2 months and a median OS of 8 months	Hypokalemia, hypomagnesemia, nausea, diarrhea	[107]
VEN + Ibrutinib + Obinuzumab	BTK CD20	recurrent MCL	NCT02558816	I/II	2-year PFS rate was 69.5% and OS rate was 68.6%	Neutropenia, diarrhea	[108]

*FL* follicular lymphoma, *MCL* mantle-cell lymphoma, *DLBCL* diffuse large B-cell lymphoma, *VEN* Venetoclax, *BTK* Bruton’s tyrosine kinase, *CR* complete remission, *OS* overall survival, *ORR* overall response rate, *PFS* progression-free survival

BTK inhibitors, such as Ibrutinib, have strong immunotherapeutic effects on NHL patients [99]. Previous preclinical studies have shown that BTK inhibition enhances mitochondrial BCL-2 dependence, thereby enhancing the ability of a BCL-2 inhibitor to kill cancer cells [66]. Therefore, several clinical trials of Ibrutinib combined with Venetoclax in the treatment of lymphoma have been conducted. In 2018, Tam CS et al. focused on the efficacy and safety of the Ibrutinib combined with Venetoclax in MCL patients with high-risk characteristics. The CR rate at week 16 was 62%, with an overall CR rate of 71%. Common adverse events included diarrhea (83%), fatigue (75%), and nausea or vomiting (71%) (NCT02471391) [100]. The SYMPATICO Phase III clinical trial confirmed that Ibrutinib and Venetoclax are efficacious in R/R MCL patients, with a median ORR of 81% and a CR rate of 62% after 31 months of follow-up, without an increase in adverse effects. (NCT03112174) [101]. Although Ibrutinib combined with Venetoclax has shown promising efficacy in MCL patients, dose-dependent hyperleukocytosis can occur, significantly increasing the risk of fatal hyperkalemia [102]. A dose study was subsequently conducted to determine the optimal dose combination. This study revealed that 200 mg of Venetoclax and 420 mg of Ibrutinib were the optimal dose combination, with an ORR of 93.8% and a dose-limiting toxicity incidence of 6.2% (NCT02419560) [103]. In addition, a study revealed immune subpopulation changes in NHL patients treated with the combination of Venetoclax and Ibrutinib, with the most significant changes being in CD4 + and CD8 + memory T cells and NK cells, indicating that the combination of these two targeted therapies may have a beneficial effect on immune recovery [104]. In summary, the above studies indicate that Ibrutinib combined with Venetoclax is a promising and feasible treatment option for R/R MCL patients.

In addition, research has confirmed that combination therapy with Ibrutinib and Rituximab is safe and shows antitumor activity [105]. Therefore, a combination therapy regimen of Venetoclax with BET inhibitors and an anti-CD20 mAb has emerged. In 2021, Dickinson M et al. evaluated the efficacy and safety of Venetoclax combined with the novel BET inhibitor RO6870810 (RO) and Rituximab in R/R DLBCL patients. The total effective rate was 38.5%, and the CR rate was 20.5%. In terms of safety, the most common Grade 3 or 4 adverse events were neutropenia (28%), anemia (23%) and thrombocytopenia (23%) (NCT03255096) [106]. The latest Phase Ib clinical study published by Petrillo A et al. in 2022 revealed that Venetoclax combined with Ibrutinib and Rituximab for R/R DLBCL patients with high-risk characteristics resulted in a median PFS of 2 months and a median OS of 8 months. The most common adverse events were hypokalemia (33%), hypomagnesemia (33%), nausea (33%), diarrhea (22%), and vomiting (22%) [107]. The OAsIs trial (NCT02558816) is a single-arm multicenter prospective Phase I/II trial that evaluated the efficacy and safety of Venetoclax combined with Ibrutinib and Obinuzumab in MCL patients. The 2-year PFS rate was 69.5%, and the regimen was tolerated well [108]. In summary, the above studies indicate that the combination of Venetoclax, anti-CD20 mAb and BTK inhibitors is feasible, has moderate activity and is safe for NHL patients. In the future, we should explore more reasonable combination plans to further enhance the efficacy.

CAR-T cells are a new type of therapeutic T-cell that can specifically kill tumor cells. Immune cell adoptive therapy, represented by anti-CD19 CAR-T cells, has also achieved good efficacy in NHL patients [109]. At present, relatively few clinical trials of CAR-T cells combined with Venetoclax have been conducted in NHL patients. Wei YH et al. reported the case of a Stage IV R/R DLBCL patient who developed an abdominal wall mass after receiving CAR-T-cell therapy. However, the size of the abdominal wall mass significantly decreased after continuous administration of Ibrutinib and Venetoclax. Therefore, the possibility that Ibrutinib and venetoclax have synergistic effects on CAR-T-cell therapy cannot be completely ruled out [110]. Scholars at the University of Pennsylvania Perelman School of Medicine have demonstrated synergistic antitumor effects in NHL patients by combining CAR-T-19 with Venetoclax. However, a higher dose of Venetoclax may increase CAR-T-cell toxicity [111].

B-cell lymphoma cell lines express high levels of CD47, and DLBCL patients with high CD47 expression have poor clinical prognoses. Therefore, the use of anti-CD47 mAbs may be a promising strategy for lymphoma treatment. Studies have shown that combination therapy with the anti-CD47 mAb TJC4 and Venetoclax significantly enhances synergistic antitumor properties in B-cell lymphoma. This finding suggests that TJC4 plus Venetoclax is a promising therapeutic, while inhibiting BCL-2 and CD47 may represent a new treatment mode for B-cell NHL patients [112].

## Conclusions

In this review, we discussed the drug discovery process, current clinical application status, and resistance and tolerance issues associated with BCL-2 inhibitors. We emphasized their important role in the development and maintenance of the immune response and proposed that the combination of BCL-2 inhibitors with immunotherapy may be one of the most promising treatment methods for hematologic malignancies. Specifically, this combination therapy can achieve long-term remission and prevent recurrence through immunotherapy. Moreover, it can increase the ability of BCL-2 inhibitors to kill tumor cells, thereby reducing the chances of tumor escape and ultimately reducing treatment costs. However, current clinical data concerning the use of BCL-2 inhibitors combined with immunotherapy remain limited, and large-scale clinical trials are needed. In addition, the authors believe that four questions regarding combination therapy warrant further exploration. First, the impact and mechanism of BCL-2 inhibitors on the number and function of tumor-infiltrating immune cells (such as various B and T cells, dendritic cells, and natural killer cells) are unclear. Second, the optimal timing and dosage for implementing immunotherapy remain to be identified. The effectiveness of each combination therapy and their long-term impacts on patient prognosis need to be elucidated. Fourth, the mechanism of combination therapy has not been identified. These questions still need extensive basic and prospective clinical research, and future research should primarily focus on exploring more reasonable combination plans, optimal treatment timing and optimal dosages.

## Data Availability

No datasets were generated or analysed during the current study.
